# Enzymatic Sialylation of IgA1 *O*-Glycans: Implications for Studies of IgA Nephropathy

**DOI:** 10.1371/journal.pone.0099026

**Published:** 2014-06-11

**Authors:** Kazuo Takahashi, Milan Raska, Milada Stuchlova Horynova, Stacy D. Hall, Knud Poulsen, Mogens Kilian, Yoshiyuki Hiki, Yukio Yuzawa, Zina Moldoveanu, Bruce A. Julian, Matthew B. Renfrow, Jan Novak

**Affiliations:** 1 Department of Microbiology, University of Alabama at Birmingham, Birmingham, Alabama, United States of America; 2 Department of Medicine, University of Alabama at Birmingham, Birmingham, Alabama, United States of America; 3 UAB Biomedical FT-ICR MS Laboratory, Department of Biochemistry and Molecular Genetics, University of Alabama at Birmingham, Birmingham, Alabama, United States of America; 4 Department of Nephrology, Fujita Health University School of Medicine, Toyoake, Japan; 5 Faculty of Medicine and Dentistry, Department of Immunology, Palacky University in Olomouc, Olomouc, Czech Republic; 6 Department of Biomedicine, Aarhus University, Aarhus, Denmark; 7 Fujita Health University School of Health Sciences, Toyoake, Japan; Institut national de la santé et de la recherche médicale (INSERM), France

## Abstract

Patients with IgA nephropathy (IgAN) have elevated circulating levels of IgA1 with some *O*-glycans consisting of galactose (Gal)-deficient *N*-acetylgalactosamine (GalNAc) with or without *N*-acetylneuraminic acid (NeuAc). We have analyzed *O*-glycosylation heterogeneity of naturally asialo-IgA1 (Ale) myeloma protein that mimics Gal-deficient IgA1 (Gd-IgA1) of patients with IgAN, except that IgA1 *O*-glycans of IgAN patients are frequently sialylated. Specifically, serum IgA1 of healthy controls has more α2,3-sialylated *O*-glycans (NeuAc attached to Gal) than α2,6-sialylated *O*-glycans (NeuAc attached to GalNAc). As IgA1-producing cells from IgAN patients have an increased activity of α2,6-sialyltransferase (ST6GalNAc), we hypothesize that such activity may promote premature sialylation of GalNAc and, thus, production of Gd-IgA1, as sialylation of GalNAc prevents subsequent Gal attachment. Distribution of NeuAc in IgA1 *O*-glycans may play an important role in the pathogenesis of IgAN. To better understand biological functions of NeuAc in IgA1, we established protocols for enzymatic sialylation leading to α2,3- or α2,6-sialylation of IgA1 *O*-glycans. Sialylation of Gal-deficient asialo-IgA1 (Ale) myeloma protein by an ST6GalNAc enzyme generated sialylated IgA1 that mimics the Gal-deficient IgA1 glycoforms in patients with IgAN, characterized by α2,6-sialylated Gal-deficient GalNAc. In contrast, sialylation of the same myeloma protein by an α2,3-sialyltransferase yielded IgA1 typical for healthy controls, characterized by α2,3-sialylated Gal. The GalNAc-specific lectin from *Helix aspersa* (HAA) is used to measure levels of Gd-IgA1. We assessed HAA binding to IgA1 sialylated at Gal or GalNAc. As expected, α2,6-sialylation of IgA1 markedly decreased reactivity with HAA. Notably, α2,3-sialylation also decreased reactivity with HAA. Neuraminidase treatment recovered the original HAA reactivity in both instances. These results suggest that binding of a GalNAc-specific lectin is modulated by sialylation of GalNAc as well as Gal in the clustered IgA1 *O*-glycans. Thus, enzymatic sialylation offers a useful model to test the role of NeuAc in reactivities of the clustered *O*-glycans with lectins.

## Introduction

Glycosylation is one of the most common post-translational modifications of proteins; about half of mammalian proteins are glycosylated. Notably, immunoglobulins and other glycoproteins may be abnormally glycosylated in patients with autoimmune and chronic inflammatory disorders, infectious diseases, or cancer [1]–[10]. Consequently, biological functions of differentially glycosylated glycoproteins in health and disease are of growing interest in biomedical research [11].

Some glycoproteins have clustered sites of *O*-glycosylation in the segments rich in serine (Ser) and threonine (Thr). Mucins, such as membrane-associated MUC1 or secreted mucins, are prototypes of heavily *O*-glycosylated proteins. The initial step in mucin-type *O*-glycosylation is the transfer of *N*-acetylgalactosamine (GalNAc) to Ser/Thr residues catalyzed by UDP-GalNAc-polypeptide *N*-acetylgalactosaminyltransferases (GalNAc-Ts). The attached GalNAc, also called Tn antigen, can be extended by core 1 β1,3-galactosyltransferase (C1GalT1) that adds galactose (Gal) to GalNAc to form the core 1 structure (GalNAc-Gal disaccharide, called T antigen). An α2,6-sialyltransferase (ST6GalNAc; in human B cells it is exclusively ST6GalNAcII that has similar specificity as the ST6GalNAcI isoform) can produce sialylated GalNAc, called sialyl-Tn antigen, by adding *N*-acetylneuraminic acid (NeuAc) residue to GalNAc, whereas Gal can be sialylated by α2,3-sialyltransferases (*e.g.,* ST3Gal1) to form sialyl-T antigen. Sialic acids occupy the terminal positions of many glycan chains of glycoproteins and contribute to a wide variety of biological functions and disease states [12], [13]. Abnormalities in mucin *O*-glycosylation, including terminal sialylation, are common in some types of cancer [10]. Increased amounts of sialyl-Tn and sialyl-T antigens have been reported in cancer cells [10], [14]–[19], with sialylated *O*-glycans being associated with higher growth rates [20] and the metastatic process [21], [22] and interactions with cell-surface receptors [23].

**Figure 1:**
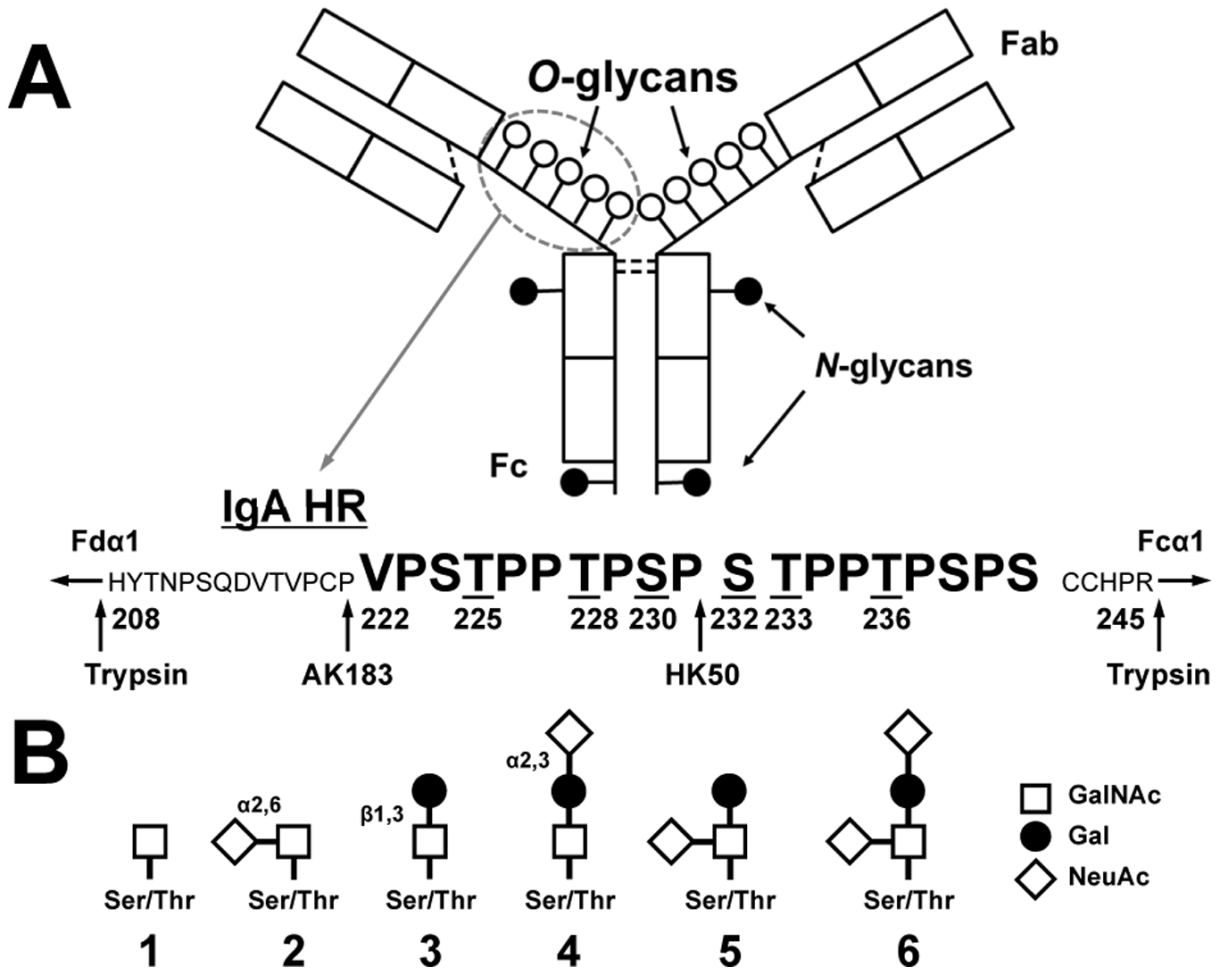
Structure of IgA1 and the hinge-region (HR) amino-acid sequence. (A) Monomeric IgA1 and its HR with nine possible sites of *O*-glycan attachment and the Fc portion of heavy chain with two *N*-glycans. Underlined serine (S) and threonine (T) residues in HR are frequently glycosylated [26], [30], [31]. Arrows show cleavage sites of trypsin and two IgA-specific proteases (from *Clostridium ramosum* AK183 and *Haemophilus influenzae* HK50). (B) *O*-glycan variants of circulatory IgA1∶1, Tn antigen; 2, sialyl-Tn antigen; 3, T antigen; 4, α3-sialyl-T antigen; 5, α6-sialyl-T antigen; 6, disialyl-T antigen. Abbreviations: GalNAc, *N*-acetylgalactosamine; Gal, galactose; NeuAc, *N*-acetylneuraminic acid.

Human IgA is represented by two structurally and functionally distinct subclasses, IgA1 and IgA2 [24]. Notably, IgA1, but not IgA2, possesses a 19-amino-acid hinge region (HR) with 9 potential *O*-glycosylation sites; 3 to 6 core 1 *O*-glycans are attached per HR [25]–[31] (Figure 1A,B). Primary IgA nephropathy (IgAN), the most common type of primary glomerulonephritis worldwide, is an immune-complex-mediated disease characterized by the presence of glomerular IgA-containing immunodeposits [32]–[36]. These deposits may be derived from IgA1-containing circulating immune complexes (CIC), often present at increased levels in patients with IgAN [37]–[43]. IgA1-containing CIC in patients with IgAN are characterized by Gal-deficient HR *O*-linked glycans of IgA1 (Gd-IgA1) [40], [41], [43]. These Gal-deficient *O*-glycans with terminal or sialylated GalNAc are recognized by anti-glycan antibodies, resulting in production of nephritogenic immune complexes that may deposit in the glomeruli, activate mesangial cells, and induce tissue injury [41], [43]–[45]. It has been shown that IgA1-producing cells from IgAN patients are responsible for the production of Gd-IgA1 due to the altered expression and activity of specific glycosyltransferases: decreased for C1GalT1 and elevated for ST6GalNAcII. Consequently, in IgAN patients the circulating levels of Gd-IgA1 with sialylated and terminal GalNAc are elevated. There are two possible mechanisms for the increased amount of sialyl-Tn antigen in IgA1 HR *O*-glycans in IgAN. In the first, augmented addition of NeuAc to GalNAc prevents further a later addition of Gal (premature sialylation); we have shown that greater activity of ST6GalNAcII is critical for the production of Gd-IgA1 [46]–[48]. Alternatively, Gal-deficient GalNAc residues may be due only to decreased C1GalT1 activity and the oversialylation of GalNAc residues would thus be a consequence of inefficient galactosylation.

Studies of sialylation of the IgA1 *O*-glycans in IgAN reported variable findings, ranging from increased [40], [41], [47], [49]–[51] to decreased [52]–[56] sialylation. Several groups have examined the role of sialylated IgA1 *O*-glycans in mesangial deposition; however, results of these studies have been inconclusive. Some studies suggested that oversialylation of IgA1 increased the negative charge of the molecule and thus increased the affinity of such IgA1 to bind mesangial cells [49], [51], [57]. In contrast, others reported that the decreased amount of NeuAc and Gal in IgA1-HR *O*-glycans enhanced affinity to extracellular matrix proteins in the mesangium [58]–[61]. Notably, two studies of IgA1 eluted from isolated glomeruli have identified less NeuAc in the mesangial IgA1 that was enriched for Gd-IgA1 [62], [63]. Furthermore, serum IgA1 from healthy controls has more α2,3-sialylated *O*-glycans than α2,6-sialylated *O*-glycans [31]. Thus, we speculate that distribution and sites of attachment of NeuAc in IgA1 *O*-glycans may play an important role in the pathogenesis of IgAN, as IgA1-HR *O*-glycans may have both α2,3- and α2,6-linked NeuAc.

To better assess the biological importance of NeuAc in the IgA1 HR, we established protocols for enzymatic sialylation leading to α2,6- or α2,3-sialylation of GalNAc and Gal, respectively. Enzymatic sialylation of terminal GalNAc of Gal-deficient asialo-IgA1 (Ale) myeloma protein generated sialylated IgA1 that mimics the Gal-deficient IgA1 glycoforms in patients with IgAN, characterized by α2,6-sialylated GalNAc. We also used the same IgA1 myeloma protein, as it has some of the clustered *O*-glycans with Gal [30], as an acceptor for α2,3-sialylation of Gal residues and generated IgA1 with α2,3-sialylated Gal typical for healthy controls. Surprisingly, reactivity with *Helix aspersa* agglutinin (HAA), which is specific for terminal GalNAc and is used in ELISA to measure levels of Gd-IgA1 [40], [41], [64], markedly decreased not only after α2,6-sialylation of GalNAc but also after α2,3-sialylation of Gal in IgA1 HR *O*-glycans. This finding is, we believe, the first demonstration that binding of GalNAc-specific lectin is modulated by sialylation of Gal-containing glycans in the clustered *O*-glycans of IgA1 with terminal GalNAc. Thus, this experimental approach is useful for testing the effects of NeuAc in clustered IgA1 *O*-glycans on lectin recognition.

## Materials and Methods

### Recombinant Glycosyltransferases

Soluble forms of recombinant human GalNAc-T2 and ST6GalNAcI were produced in insect cells Sf9 or human HEK 293T cells [65]. Recombinant ST3Gal1 was purchased from Calbiochem (La Jolla, CA).

### Acceptors for Enzyme Reactions

A panel of synthetic HR (sHR) (glyco)peptides with no GalNAc, a single GalNAc residue at five different sites, or five GalNAc residues [28] was used as enzyme acceptors for GalNAc-T2. We confirmed that preexisting sites of glycosylation on the sHR glycopeptides did not affect kinetics of the GalNAc-T2 reactions (Figure S1). Thus, sHR peptide, corresponding to the amino-acid sequence of the human IgA1 HR, was synthesized by and purchased from Bachem (Torrance, CA). The following sHR peptide was used as the acceptor for GalNAc-T2: VPSTPPTPSPSTPPTPSPSCCHPR-OH. The enzyme reaction mixture contained 25 mM Tris-HCl (pH 7.4), 5 mM MnCl_2_, 250 µM UDP-GalNAc (Sigma, St. Louis, MO), 15 µM acceptor sHR substrate or sHR glycopeptides, and the purified enzyme in a final volume of 25 µL. The reaction mixture was incubated at 37°C, samples were collected at different time points, and the reactions were stopped by boiling. Recombinant GalNAc-T2 added 3 to 7 GalNAc residues to sHR in 15 min. The polymeric form of IgA1 (Ale) myeloma protein had been previously isolated from plasma of a patient with multiple myeloma [47]. Briefly, the plasma sample was precipitated with ammonium sulfate (50% saturation) and dissolved in phosphate buffer, and IgG and IgM were removed by affinity chromatography with protein G and anti-human IgM antibodies, respectively [66]. Next, size-exclusion chromatography on a column of Ultrogel AcA22 (Amersham Biosciences, Piscataway, NJ) was used to isolate polymeric IgA1. The final purification step included FPLC separation of the polymeric form of IgA1 on a column of Sephacryl 300. We previously reported that the IgA1 (Ale) myeloma protein used in this study was naturally without sialic acid on *O*-glycans [30].

### Sialyltransferase Reactions

sHR (15 µM) with 3 to 7 GalNAc residues or 2 µg of purified IgA1 (Ale) myeloma protein was incubated with 2 µl of ST3Gal1 or ST6GalNAcI overnight at 37°C in a total volume of 20 µl reaction buffer (50 mM MES buffer, pH 6.5, 2 mM CaCl_2_, 2 mM MnCl_2_, 10 mM MgCl_2_, 5 mM CMP-NeuAc). The sialyltransferase reaction with the sHR acceptor with 3 to 7 GalNAc residues was terminated by boiling. The enzyme reaction with IgA1 myeloma protein as acceptor was terminated by snap-freezing the samples at −80°C [67].

### SDS-PAGE

IgA1 (Ale) myeloma protein starting samples and enzymatically sialylated IgA1 samples were cleaved with IgA-specific protease from *Clostridium ramosum* AK183 (recombinant enzyme produced in *Escherichia coli*) and were separated by SDS-PAGE using 4–15% gradient slab gels (Bio-Rad, Hercules, CA) under reducing conditions; the proteins were detected by silver staining.

### HAA Lectin-ELISA

F(ab’)_2_ fragment of goat anti-human IgA (Jackson ImmunoResearch, West Grove, PA) at a concentration of 3 µg/ml was coated onto the wells of Costar 96-well plates (Corning Inc., Corning, NY). Plates were blocked overnight at 4°C with 2% BSA (Sigma-Aldrich, St. Louis, MO) in PBS containing 0.05% Tween 20 (v/v). Samples of IgA1 diluted in the blocking buffer were added to each well and incubated overnight at 4°C. For neuraminidase treatment, the captured IgA was subsequently desialylated by treatment for 3 h at 37°C with 10 mU/ml neuraminidase from *Vibrio cholerae* (Roche, Basel, Switzerland) in 10 mM sodium acetate buffer, pH 5 [40]. Samples were analyzed with and without neuraminidase treatment. Samples were then incubated for 3 h at 37°C with GalNAc-specific biotinylated HAA lectin (Sigma-Aldrich) diluted 1∶500 in blocking buffer [45], [47], [64], [66]. The bound lectin was detected with avidin-horseradish peroxidase conjugate, and the reaction was developed. HAA binding to IgA1 was expressed relative to the standard IgA1 (Ale) myeloma protein [45], [47].

### Proteolytic Release of IgA-HR Glycopeptides

IgA1 proteins were treated with an IgA-specific protease (from *Clostridium ramosum* AK183 or from *Haemophilus influenzae* HK50 that differ in the cleavage site; see Figure 1A), followed by trypsin cleavage, to release IgA1 HR glycopeptides [30], [31]. The digests were desalted by use of a C18 spin column (Pierce, Rockford, IL) before mass spectrometric (MS) analyses.

### High-resolution MS Analysis

On-line LC was performed by use of an Eksigent MicroAS autosampler and 2D LC nanopump (Eksigent, Dublin, CA). One-hundred-fifty nanograms of digested IgA1 were loaded onto a 100-µm-diameter, 11-cm column pulled tip packed with Jupiter 5-µm C18 reversed-phase beads (Phenomenex, Torrance, CA). The digests were then eluted with an acetonitrile gradient from 5 to 30% in 0.1% formic acid over 50 min at 650 nL min^−1^. Linear quadrupole ion trap Orbitrap Velos (Orbitrap) mass spectrometry (Thermo Fisher Scientific, San Jose, CA) parameters were as described previously [30], [31]. Briefly, Orbitrap parameters were set to normal mass range (MS1, 300< *m/z* <1800) with a 50,000 resolution scan followed by five data-dependent collision-induced dissociation tandem MS scans per cycle in profile mode. Dynamic exclusion was set to exclude ions for 2 min after a repeat count of three within a 45-sec duration. All spectra were analyzed by use of Xcalibur Qual Browser 2.1 (Thermo Fisher Scientific) software. Identified IgA1 HR *O*-glycopeptides were checked against theoretical values by use of GlycoMod tool (http://www.expasy.org). Known IgA1 HR amino-acid sequences produced by the combination of IgA-specific protease+trypsin digestions were inputted with trypsin enzyme and 0 missed cleavage sites. Hexose, *N*-acetylhexosamine (HexNAc) and NeuAc monosaccharide residues were all selected as possible (variable) additions to the IgA HR peptides with a mass tolerance of 10 ppm.

## Results

### Enzymatic α2,6 Sialylation of Synthetic HR (sHR) with GalNAc Residues

**Figure 2:**
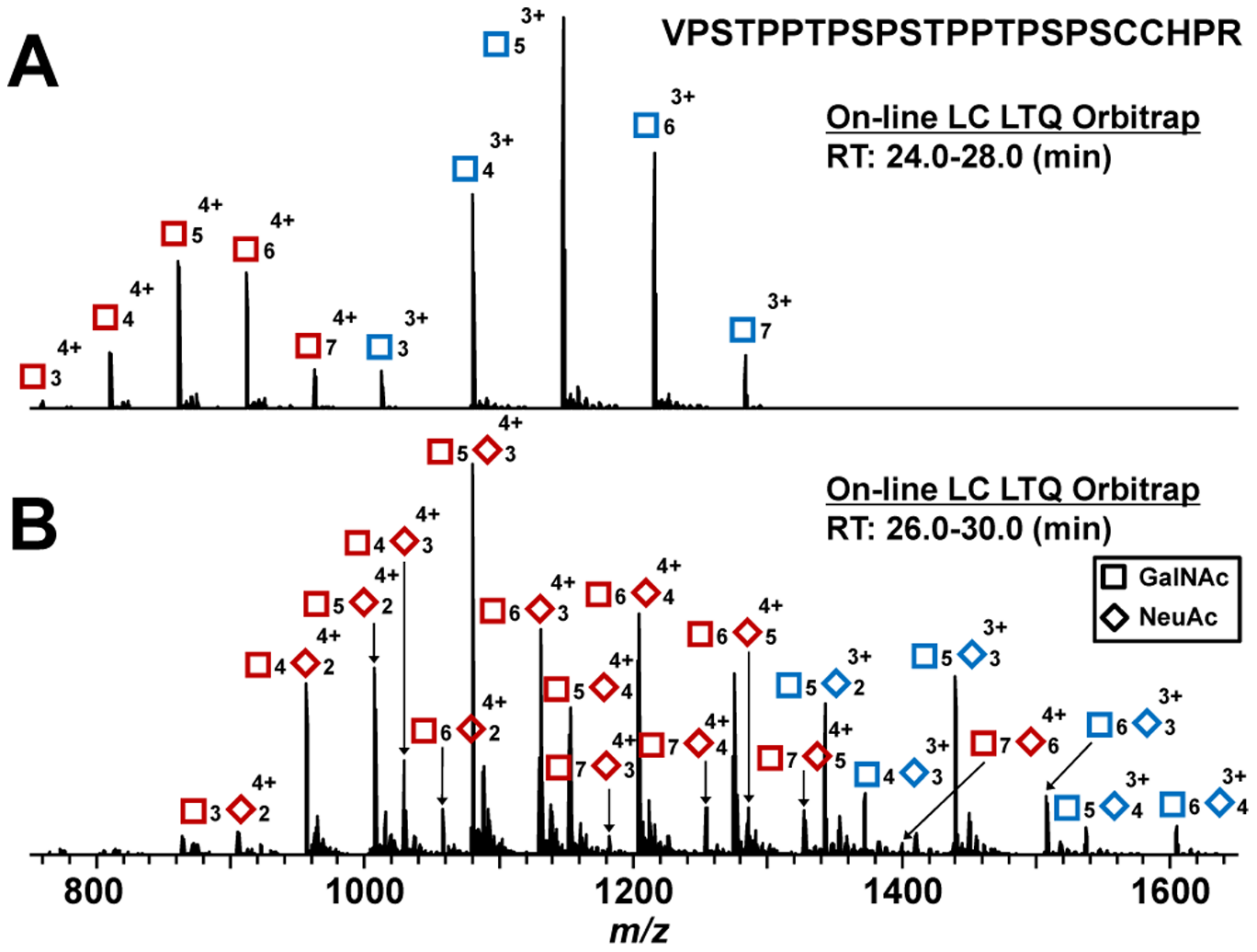
MS analysis of sialylation of GalNAcosylated synthetic HR glycopeptides (sHR) by ST6GalNAcI. (A) Acceptor sHR glycopeptides for ST6GalNAcI with 3 to 7 GalNAc residues were generated by recombinant GalNAc-T2 after a 15-min reaction. (B) sHR glycopeptides produced after over-night reaction with ST6GalNAcI. Symbols: GalNAc, square; NeuAc, diamond. Red symbols: glycopeptides ionized as 4+ ions; Blue symbols: glycopeptides ionized as 3+ ions.

We successfully produced a recombinant soluble form of enzymatically active ST6GalNAcI. This enzyme can sialylate GalNAc of glycoproteins, similarly as does the ST6GalNAcII isoform expressed in human IgA1-producing cells. sHR glycopeptide with 3 to 7 GalNAc residues, generated from sHR in GalNAc-T2 reaction for 15 min, was used as an acceptor substrate for ST6GalNAcI (Figure 2A). ST6GalNAcI added three to six NeuAc residues to sHR peptides with three to seven GalNAc residues, with at least one GalNAc remaining without NeuAc (Figure 2B).

### Enzymatic α2,3 Sialylation of Native IgA1 Protein

**Figure 3:**
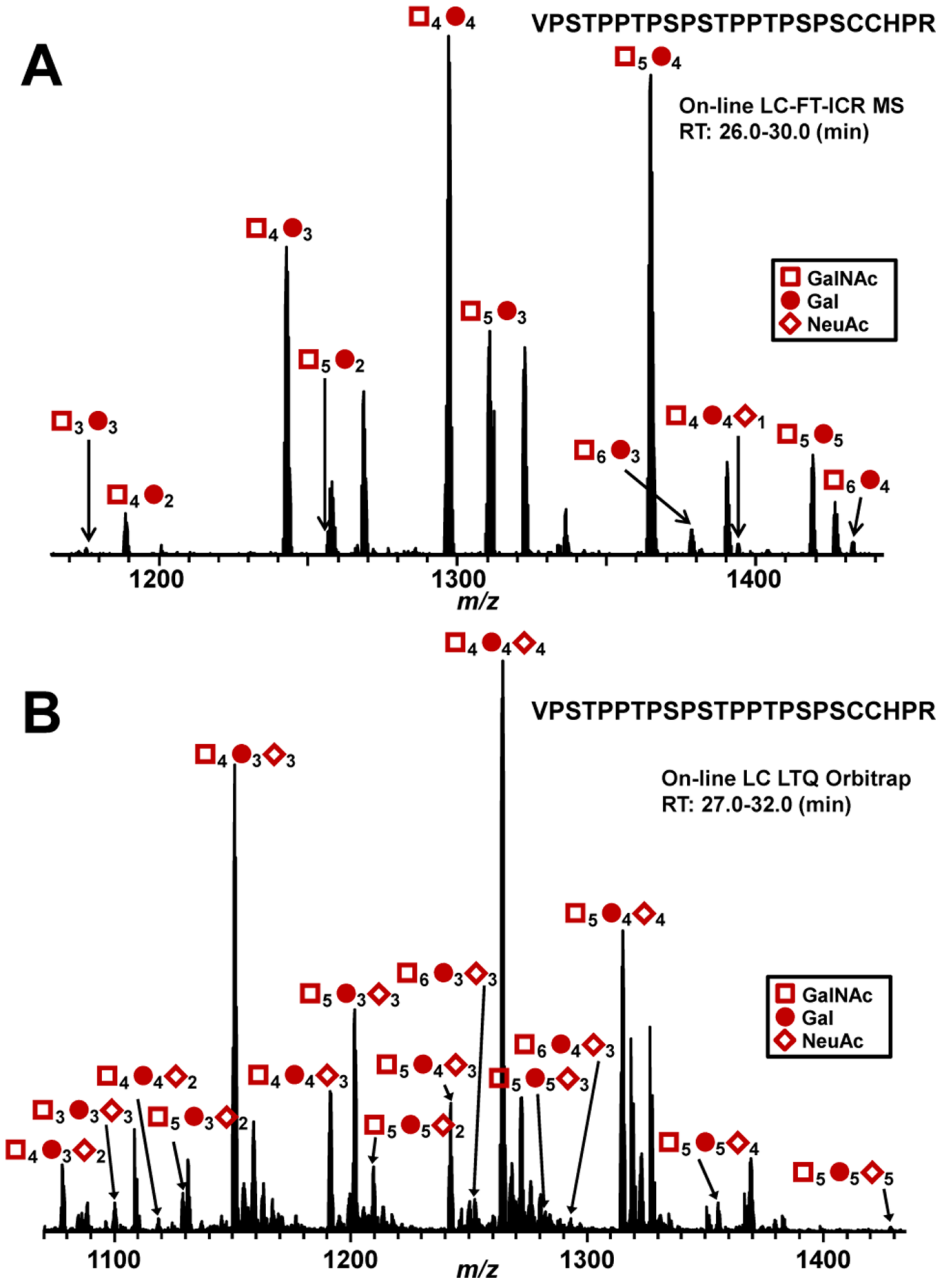
MS analysis of sialylation by ST3Gal1 of IgA1 myeloma protein that is naturally sialic-acid-deficient. (A) HR *O*-glycan profile of IgA1 (Ale) myeloma protein. The number of *O*-glycans was assigned based on the masses of the amino-acid sequence, GalNAc (empty squares), Gal (full circles), and NeuAc (full diamonds). The *O*-glycans of the protein are minimally sialylated. All HR *O*-glycoforms are ionized as triply charged ions. (B) HR *O*-glycan profile of IgA1 (Ale) myeloma protein after over-night sialylation reaction with ST3Gal1. The enzyme added NeuAc residues to the *O*-glycans of Ale myeloma protein. All HR *O*-glycoforms are ionized as quadruply charged ions.

**Figure 4:**
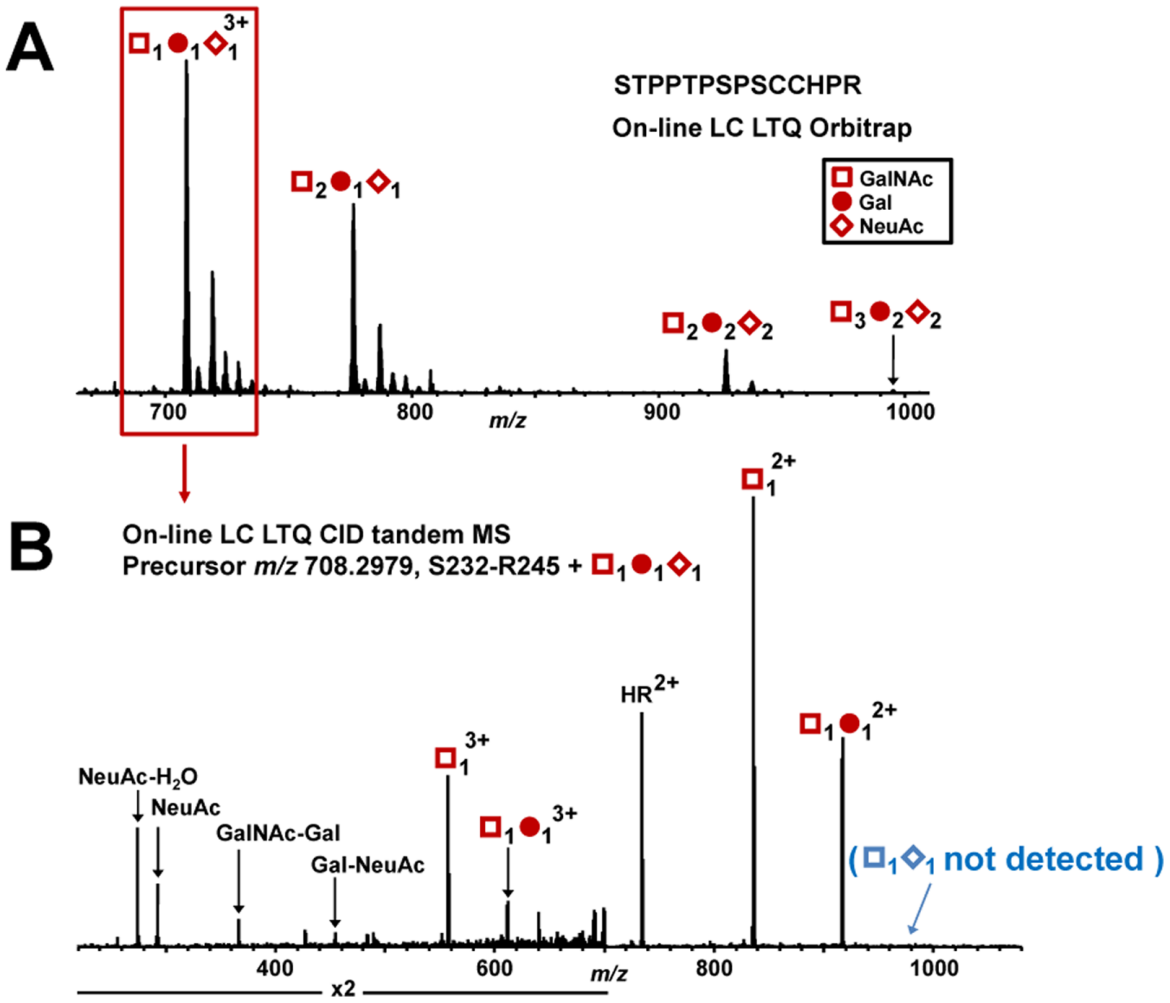
LC-CID fragmentation of IgA1 Ser^232^-Arg^245^ (HR) with GalNAc_1_Gal_1_NeuAc_1_. (A) Ser^232^-Arg^245^ HR *O*-glycan profile of IgA1 (Ale) myeloma protein enzymatically sialylated with ST3Gal1. (B) LC-CID tandem MS spectrum of Ser^232^-Arg^245^ with GalNAc_1_Gal_1_NeuAc_1_. Absence of sialylated GalNAc (shown in blue parenthesis) indicates that NeuAc is attached to Gal (by an α2,3-linkage) in the precursor ion. Additionally, the presence of the Gal-NeuAc oxonium ion confirms the attachment of the NeuAc to Gal.

ST3Gal1 adds sialic acid to the Gal residue of T antigen (GalNAc-Gal). To determine whether recombinant ST3Gal1 adds NeuAc residues to clustered *O*-glycans of IgA1, we used naturally sialic-acid-deficient IgA1 (Ale) myeloma protein, with three to six *O*-glycans with up to five T antigens, as an acceptor substrate (Figure 3A). Our analysis showed that ST3Gal1 added NeuAc residues to all five T antigens in the clustered HR *O*-glycans (Figure 3B). IgA-specific protease from *Haemophilus influenzae* HK50 and trypsin produced N-terminal 24-mer glycopeptides (His^208^-Pro^231^) and C-terminal 14-mer glycopeptides (Ser^232^-Arg^245^) (Figure 1A). To determine whether ST3Gal1 added NeuAc residue to only Gal residues, Ser^232^-Arg^245^ HR glycopeptide with GalNAc_1_Gal_1_NeuAc_1_ was subjected to online liquid chromatography (LC) collision-induced dissociation (CID) tandem mass spectrometry (MS/MS) (Figure 4A,B). Primary absence of NeuAc in the precursor ion indicated that NeuAc was attached to Gal to form a linear trisaccharide (GalNAc-Gal-NeuAc) (Figure 4B). Additionally, the presence of a Gal-NeuAc oxonium ion further confirmed that the addition was the linear trisaccharide. We have previously analyzed IgA1-HR *O*-glycoforms of normal human serum IgA1 and found that most T antigens was α2,3-sialylated [31]. Thus, such an enzymatically α2,3-sialylated IgA1 mimics IgA1 from normal human serum.

### Model of Distinct α2,3/α2,6-sialylated IgA1 HR *O*-glycoforms

**Figure 5:**
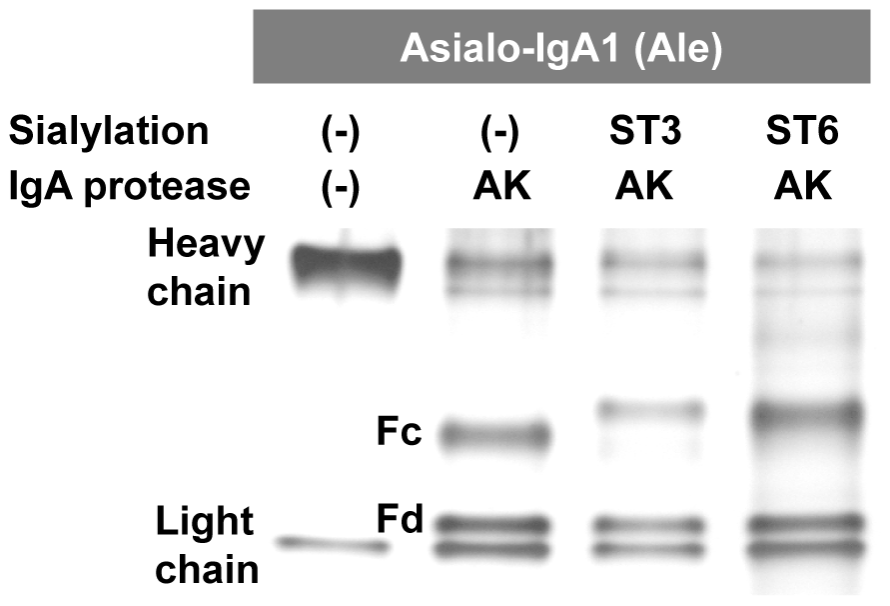
SDS-PAGE of IgA1 (Ale) myeloma protein. IgA1 proteins were separated by SDS-PAGE under reducing conditions and the protein bands were silver stained. IgA1 was untreated or sialylated with ST3Gal1 (ST3) or ST6GalNAcI (ST6) sialyltransferases and the Fc and Fd fragments of the heavy chains were generated using IgA-specific protease from *Clostridium ramosum* AK183 (see Fig. 1). Mobility change of Fc fragments after sialyltransferase reactions confirmed sialylation of HR *O*-glycans.

To establish protocols for enzymatic sialylation of either Gal or GalNAc in IgA1-HR clustered *O*-glycans, asialo-IgA1 (Ale) myeloma protein was sialylated using ST3Gal1 or ST6GalNAcI, respectively. Significant changes in SDS-PAGE mobility after sialyltransferase reactions of IgA1 Fc fragment produced by IgA-specific protease from *Clostridium ramosum* AK183 indicated that both ST3Gal1 and ST6GalNAcI added NeuAc to IgA1 HR *O*-glycans (Figure 5). Enzymatic sialylation of Gal-deficient asialo-IgA1 (Ale) myeloma protein thus generated sialylated IgA1 that mimics the Gal-deficient IgA1 glycoforms in patients with IgAN, characterized by α2,6-sialylated GalNAc, or the IgA1 typical for healthy controls, characterized by α2,3-sialylated Gal.

### Lectin Binding to Sialylated IgA1

**Figure 6:**
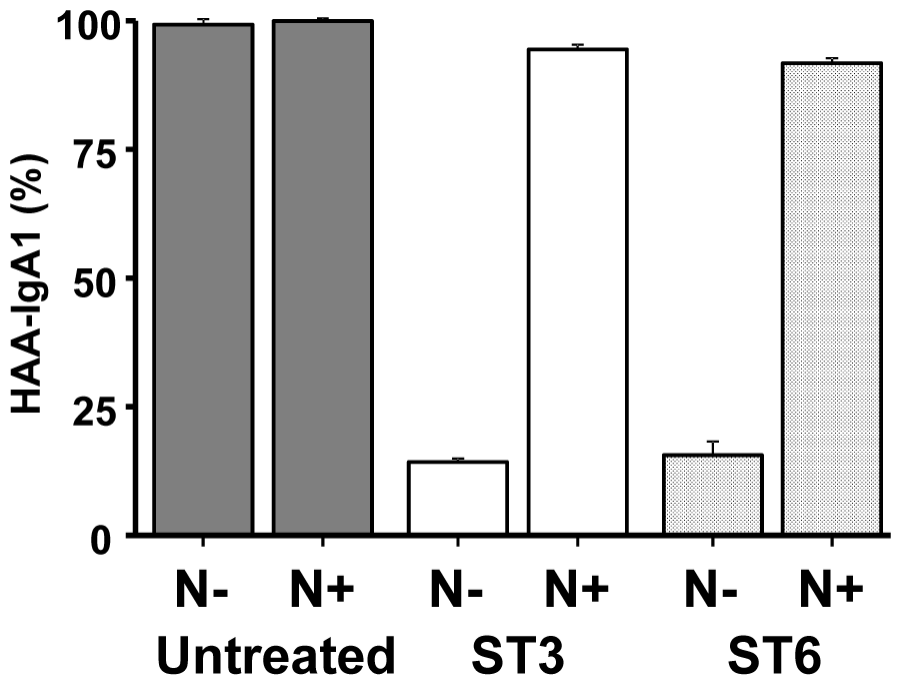
HAA reactivity of IgA1 (Ale) myeloma protein with (N+) or without (N-) neuraminidase treatment. HAA lectin binding to Gal-deficient IgA1 (Ale) in ELISA is reduced by sialylation of GalNAc as well as of Gal by specific sialyltransferases. HAA binding to an untreated IgA1 protein is set to 100%.

A GalNAc-specific lectin, HAA, is used in ELISA to measure levels of Gd-IgA1. Notably, α2,6- as well as α2,3-sialylation of IgA1 HR *O*-glycans markedly decreased reactivity with HAA (Figure 6). Neuraminidase treatment recovered the original HAA reactivity. These results thus suggest that binding of GalNAc-specific lectin is affected not only by sialylation of GalNAc but also by sialylation of Gal in the clustered *O*-glycans of Gal-deficient IgA1.

## Discussion

Patients with IgAN have elevated circulating levels of IgA1 with some *O*-glycans consisting of terminal or sialylated GalNAc. We have previously analyzed *O*-glycosylation of naturally sialic-acid-deficient IgA1 (Ale) myeloma protein that mimics the aberrant (*i.e.,* Gal-deficient) IgA1 in patients with IgAN, although HR *O*-glycans of circulatory IgA1 are frequently sialylated. It has been suggested that the anionic nature of IgA1 may promote mesangial IgA1 deposition [68], [69] and the anionic character of IgA1 is in agreement with less NeuAc in IgA1 HR *O*-glycans [47], [49]–[51]. However, IgA1 eluted from isolated glomeruli has decreased level of NeuAc compared to IgA1 in the circulation of the corresponding IgAN patients [62], [63]. These observations of altered sialylation of IgA1 *O*-glycans have become a significant area of interest in the pathogenesis of IgAN. We have reported that IgA1-producing cells from IgAN patients have increased expression of ST6GalNAcII, an isoform closely related to ST6GalNAcI, and that IgA1 secreted by IgA1-producing cells from IgAN patients contained terminal and sialylated GalNAc [47]. Furthermore, our high-resolution MS analysis showed that IgA1 from healthy controls had more α2,3-sialylated *O*-glycans than α2,6-sialylated *O*-glycans [31]. We speculate that distribution of NeuAc in IgA1 *O*-glycans may play an important role in the pathogenesis of IgAN, as IgA1-HR *O*-glycans have α2,3- as well as α2,6-linked NeuAc. To study the effect of NeuAc in the IgA1 HR on lectin binding, we established protocols for enzymatic sialylation leading to α2,3- or α2,6-sialylation of Gal and GalNAc, respectively. These protocols allow linkage-specific sialylation to assess the effect of the respective type of sialylation on IgA1 lectin recognition.

Biological roles of NeuAc in clustered *O*-glycans of IgA1 are not fully understood. NeuAc residues in IgA1 HR *O*-glycans influence the affinity of IgA1 to some receptors. For example, binding of IgA1 to asialoglycoprotein receptor (ASGP-R) is reduced by sialylation of IgA1 [70]–[75]. It also has been suggested that enhanced sialylation of IgA1 extended its half-life in the circulation due to reduced clearance [1], [49], [51].

The association of recurrent macroscopic hematuria with upper-respiratory-tract infections in IgAN patients led to the suggestion that the production of pathogenic IgA1 may be related to abnormal handling of mucosal antigens. Gd-IgA1 in the patients with IgAN is found almost exclusively in CIC bound to IgG or IgA1 antiglycan antibodies [37]–[41]. We recently have shown that these IgG antibodies recognize GalNAc-containing epitopes on the Gal-deficient HR *O*-glycans of IgA1 [43]. As to the origin of these antibodies, it has been suggested that they may primarily recognize GalNAc-containing epitopes on viruses (*e.g.,* Epstein-Barr virus) or bacteria (streptococci) and that they happen to cross-react with glycans on Gd-IgA1 [76]. As surfaces of microbes can be sialylated [77], NeuAc in IgA1-HR *O*-glycans may play an important role in the recognition by specific antibodies against the HR of Gd-IgA1. Our enzymatic sialylation protocol in conjunction with MS/MS analyses of the resultant products will be useful for the molecular studies of the glycoprotein or glycopeptide structures that may exhibit strong affinity to glycan-specific antibodies recognizing Gd-IgA1.

IgA-specific proteases are proteolytic enzymes that cleave specific peptide bonds in the human IgA HR [78]. Several species of pathogenic bacteria secrete IgA-specific proteases at mucosal sites of infection that neutralize effector functions of human IgA1 and thereby eliminate an important aspect of host defense. Thus, IgA-specific proteases are considered virulence factors, as they prevent effective IgA-mediated immune defense that requires intact IgA [78]–[80]. Importantly, some of these bacteria (e.g., *Streptococcus pneumoniae*) also secrete neuraminidase [80]–[84] that removes sialic acid in the first step of the breakdown of soluble mucins as well as cell-surface glycoconjugates [13]. It is thus conceivable that structural changes by desialylation of IgA1 may facilitate the recognition of Gd-IgA1 HR *O*-glycans by antiglycan IgG. This hypothesis may explain the association of macroscopic hematuria with upper-respiratory-tract infections. In this setting, the amount of circulating antiglycan autoantibodies presumably increases due to the infection. The antibodies bind to Gd-IgA1, resulting in formation of IgA1-IgG complexes, with subsequent renal deposition, mesangial cell activation, and glomerular injury (for review, see [36]).

We have developed a new model for sugar-specific sialylation of IgA1 *O*-glycans and showed that GalNAc recognition by HAA lectin is modulated by sialylation of not only GalNAc but also of Gal in the clustered IgA1 *O*-glycans. We envision that our enzymatic sialylation protocol will be useful for the study the biological roles of sialic acid in IgA1-HR *O*-glycans. Moreover, characterizing IgA1-HR glycoforms, including sialylation, is important for understanding the pathogenesis of IgAN and developing disease-specific biomarkers [85].

## Supporting Information

Figure S1 **The amount of GalNAc residues attached to HR acceptor substrates in the time-course study with GalNAc-T2.** The amount of GalNAc attached to acceptor substrates was calculated based on the relative abundance of each glycoform. The following HR glycopeptides with a single GalNAc residue at different sites were used as enzyme acceptors: 4-HP: VPST(GalNAc)PPTPSPSTPPTPSPS, 7-HP: VPSTPPT(GalNAc)PSPSTPPTPSPS, 9-HP: VPSTPPTPS(GalNAc)PSTPPTPSPS, 11-HP: VPSTPPTPSPS(GalNAc)TPPTPSPS, 15-HP: VPSTPPTPSPSTPPT(GalNAc)PSPS. A synthetic HR peptide and HR glycopeptide with five GalNAc residues attached were also used: HP: VPSTPPTPSPSTPPTPSPS; All-HP:VPST(GalNAc)PPT(GalNAc)PS(GalNAc)PS(GalNAc)TPPT(GalNAc)PSPS.
(TIF)
