# Correction: Enzymatic Sialylation of IgA1 *O*-Glycans: Implications for Studies of IgA Nephropathy

**DOI:** 10.1371/journal.pone.0113577

**Published:** 2014-11-13

**Authors:** The PLOS ONE Staff

The following information is missing from the funding section: grants LH11046 (Ministry of Education, Youth, and Sport, Czech Republic) and CZ.1.07/2.3.00/20.0164 European Social Fund. The correct Funding statement is: This study was supported in part by the NIH grants DK078244, GM098539, and DK082753, and grants LH11046 (Ministry of Education, Youth, and Sport, Czech Republic) and CZ.1.07/2.3.00/20.0164 European Social Fund, and by a gift from the IGA Nephropathy Foundation of America. The funders had no role in study design, data collection and analysis, decision to publish, or preparation of the manuscript.

## References

[1] Takahashi K, Raska M, Stuchlova Horynova M, Hall SD, Poulsen K, et al (2014) Enzymatic Sialylation of IgA1 *O*-Glycans: Implications for Studies of IgA Nephropathy. PLoS ONE 9(6): e99026 doi:10.1371/journal.pone.0099026 24918438 10.1371/journal.pone.0099026 PMC4053367

