# Supplementary figures and images for: Enzymatic Sialylation of IgA1 O-Glycans: Implications for Studies of IgA Nephropathy
Source: PLoS One. 2014 Jun 11;9(6):e99026. doi: 10.1371/journal.pone.0099026 (PMC4053367; doi:10.1371/journal.pone.0099026)

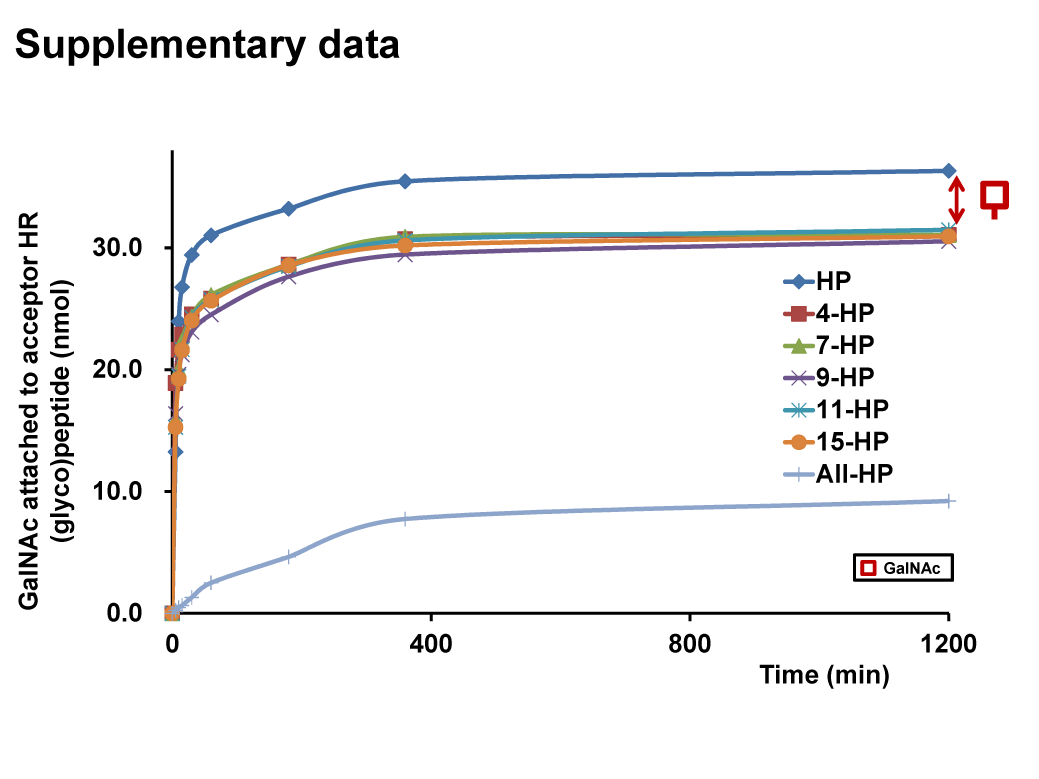

Supplement: Figure S1 — The amount of GalNAc residues attached to HR acceptor substrates in the time-course study with GalNAc-T2. The amount of GalNAc attached to acceptor substrates was calculated based on the relative abundance of each glycoform. The following HR glycopeptides with a single GalNAc residue at different sites were used as enzyme acceptors: 4-HP: VPST(GalNAc)PPTPSPSTPPTPSPS, 7-HP: VPSTPPT(GalNAc)PSPSTPPTPSPS, 9-HP: VPSTPPTPS(GalNAc)PSTPPTPSPS, 11-HP: VPSTPPTPSPS(GalNAc)TPPTPSPS, 15-HP: VPSTPPTPSPSTPPT(GalNAc)PSPS. A synthetic HR peptide and HR glycopeptide with five GalNAc residues attached were also used: HP: VPSTPPTPSPSTPPTPSPS; All-HP:VPST(GalNAc)PPT(GalNAc)PS(GalNAc)PS(GalNAc)TPPT(GalNAc)PSPS. (TIF) [file pone.0099026.s001.tif]
